# Regulation of Neuroinflammatory Signaling by PPARγ Agonist in Mouse Model of Diabetes

**DOI:** 10.3390/ijms23105502

**Published:** 2022-05-14

**Authors:** Iwona Piątkowska-Chmiel, Mariola Herbet, Monika Gawrońska-Grzywacz, Jarosław Dudka

**Affiliations:** Chair and Department of Toxicology, Faculty of Pharmacy, Medical University of Lublin, Jaczewskiego 8b Street, 20-090 Lublin, Poland; mariola.herbet@umlub.pl (M.H.); monika.gawronska-grzywacz@umlub.pl (M.G.-G.); jaroslaw.dudka@umlub.pl (J.D.)

**Keywords:** neuroinflammation, diabetes, pioglitazone, anti-inflammatory effect, exploratory behaviors, memory

## Abstract

Many relevant studies, as well as clinical practice, confirm that untreated diabetes predisposes the development of neuroinflammation and cognitive impairment. Having regard for the fact that PPAR*γ* are widely distributed in the brain and PPAR*γ* ligands may regulate the inflammatory process, the anti-inflammatory potential of the PPAR*γ* agonist, pioglitazone, was assessed in a mouse model of neuroinflammation related with diabetes. In this regard, the biochemical and molecular indicators of neuroinflammation were determined in the hippocampus and prefrontal cortex of diabetes mice. The levels of cytokines (IL-1*β*, IL-6, and TNF) and the expression of genes (*Tnfrsf1a* and *Cav1*) were measured. In addition, behavioral tests such as the open field test, the hole-board test, and the novel object recognition test were conducted. A 14-day treatment with pioglitazone significantly decreased IL-6 and TNFα levels in the prefrontal cortex and led to the downregulation of *Tnfrsf1a* expression and the upregulation of *Cav1* expression in both brain regions of diabetic mice. Pioglitazone, by targeting neuroinflammatory signaling, improved memory and exploratory activity in behavioral tests. The present study provided a potential theoretical basis and therapeutic target for the treatment of neuroinflammation associated with diabetes. Pioglitazone may provide a promising therapeutic strategy in diabetes patients with muffled of behavioral activity.

## 1. Introduction

Neuroinflammation is involved in the development and progress of a wide range of disorders of the central nervous system (CNS). It is considered as a crucial mechanism in in the pathogenesis of Alzheimer’s disease (AD) [1,2,3], Parkinson’s disease (PD) [2,3,4], epilepsy [5,6], schizophrenia [7], bipolar disorder [8], as well as depression [9,10]. Accumulative evidence suggests that there is also an undeniable relationship between the neuroinflammation and development and progression of CNS disorders in diabetes patients [11,12,13,14]. The neuronal disturbance in the course of diabetes can be associated with prolonged microglial and astrocyte activation that in response leads to the release of cytokines such as interleukin (IL)-1*β*, IL-6, tumor necrosis factor-*α* (TNF*α*), and interferon gamma (IFN*γ*), and chemokines such as ractalkine (CX3CL1) [15]. Released cytokines as small pleiotropic signaling proteins can affect the metabolism of neurotransmitters and hence modulate synaptic transmission and plasticity [16,17]. The growing body of research shows that chronic inflammation and the associated over-expression of cytokines, also accelerates neurodegeneration processes in diabetes patients [18,19,20,21]. The observed cascade of events in the course of the chronic activation of pro-inflammatory pathways in the brain of patients leads to the gradual susceptibility of neurons for damage and death, and hence the deterioration of cognitive functions (particularly in acquiring and restoring memory), psychomotor slowing [22,23,24], and the promotion of neurodegeneration [25,26,27,28,29] and depression [30]. Taking into account the dynamic worldwide growth of the incidence of diabetes (it is estimated that in 2030 the number of patients will exceed 552 million) [31,32,33] and the growing role of inflammation in the development and progression of neurological complications that occurred in this group of patients, more attention should be given to substances that can modulate the neuroinflammatory pathways. Therefore, counteracting neuroinflammation becomes one of the priority directions in modern medicine.

Pioglitazone (Pio) is a second-line drug in the treatment of type 2 diabetes, approved by the Food and Drug Administration (FDA). Pio, as an agonist of nuclear peroxisome proliferator-activated receptor (PPAR)*γ*, demonstrates broad metabolic and anti-inflammatory activities [34,35,36]. The recent studies suggest that PPAR*γ* ligands can have beneficial effect in the treatment of neuroinflammatory diseases by inhibiting the production of inflammatory factors and by the reduction of oxidative stress [37,38]. Research shows that PPAR*γ* expression occurs in multiple brain regions [39]. The activation of PPAR*γ* in the neurons improves the mitochondrial function and it leads to the degradation or inactivation of transcriptional inflammatory regulators, and the reduction of reactive oxygen species (ROS) levels [40,41,42]. Pioglitazone alleviated stroke symptoms in rats under the MCAO test as quickly as 3 days after reperfusion [43]. A positive neuroprotective effect was also confirmed by in vitro tests [42,43,44,45]. Moreover, pioglitazone used in various models of neurodegenerative diseases such as Alzheimer’s disease and Parkinson’s disease confirmed its neuroprotective action [46,47,48,49,50,51,52,53]. In Alzheimer’s disease models, it improved spatial learning, attenuated inflammation in the nervous system and tau hyperphosphorylation [54].

Studies showed that pioglitazone can inhibit inflammation and oxidative stress also in a PPAR *α*-dependent manner [55,56,57]. Researchers proved that pioglitazone can ameliorate these processes by modulating the mitochondrial functioning and/or inflammatory reactions [55,56,57,58]. Orasanu et al. [55] showed that pioglitazone can represses inflammatory responses involving endothelial VCAM-1 and hepatic IκBα in a PPAR *α*-dependent manner both in vitro and in vivo.

Furthermore, Assaf et al. [56] demonstrated the neuroprotective effects of combined PPAR*α* and *γ* agonist therapy in a mouse model of amyloidogenesis through modulation of the Wnt/beta catenin pathway. A three-week treatment led to reduced behavioral, neurochemical, and histopathological changes in the treated mice. Studies indicated that specific activators of PPAR *α* may be significant for the improvement of brain cell metabolism and cognitive function in neurodegenerative and neurodevelopmental disorders [57].

Based on the above, the aim of our study was to evaluate the effects of pioglitazone on the central nervous system of mice in a model of neuroinflammation related with diabetes. Behavioral tests (the hole-board test, the novel object recognition test, and the open field test), biochemical tests (cytokines in the serum and brain supernatants), and molecular tests (expression of the *Tnfrsf1a* and *Cav1* genes) were performed to elucidate the mechanisms of the neuroprotective action of pioglitazone.

## 2. Results

### 2.1. Effect of Pioglitazone on Exploratory Activity and Memory of Diabetic Mice

**Figure 1:**
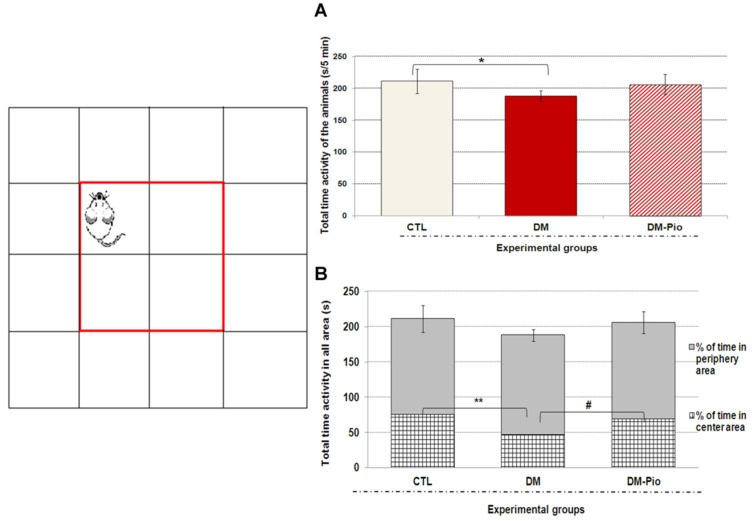
Effect of pioglitazone on exploratory activity in mice with neuroinflammation linked to diabetes (**A**,**B**). The bar height represents the mean ± SD for individual experimental groups: CTL: non-diabetic mice, DM: diabetic mice, DM-Pio: pioglitazone-treated mice [30 mg/kg, *po*]. One-way ANOVA, followed by Tukey’s post hoc test was used. * *p* < 0.05; ** *p* < 0.01 in comparison with control group. # *p* < 0.05 in comparison with diabetes group.

**Figure 2:**
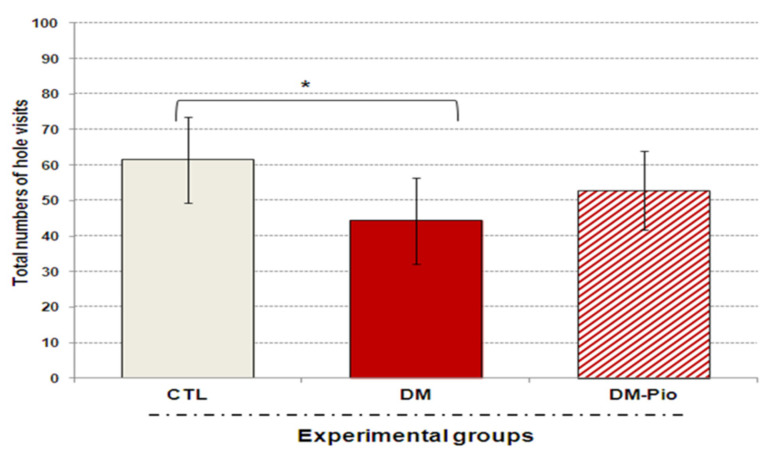
Effect of pioglitazone on exploratory activity of mice with neuroinflammation linked to diabetes. The bar height represents the mean ± SD for individual experimental groups: CTL: non-diabetic mice, DM: diabetic mice, DM-Pio: pioglitazone-treated mice [30 mg/kg, *po*]. One-way ANOVA, followed by Tukey’s post hoc test was used. * *p* < 0.05 in comparison with control group.

As shown in Figure 1 and Figure 2, a 14-day treatment with Pioglitazone (30 mg/kg *po*) had no significant effect on the exploratory activity of diabetes mice in the open-field test (OFT) and the hole-board test (HBT). The data did not show significant differences in the exploratory activity of mice, demonstrating that drug administration did not affect normal movement in adult mice. Although the animals of the treated group seemed to be more active than in the untreated group, what was shown in both tests was that the statistical analysis of the results showed no significant differences between these groups (Figure 1A and Figure 2; *p* > 0.05). Interestingly, during the open-field test (OFT) treated animals remained more often in the central part of the apparatus than untreated animals. Animals spent almost 10% more time exploring the environment in the central part of the arena than diabetic mice (Figure 1B, # *p* < 0.05 F_(3.32)_ = 7.268). Moreover, % of time spent in the center and periphery of the arena by the treated animals was comparable to the percentage of animal activity in the above-mentioned areas in the control group.

**Figure 3:**
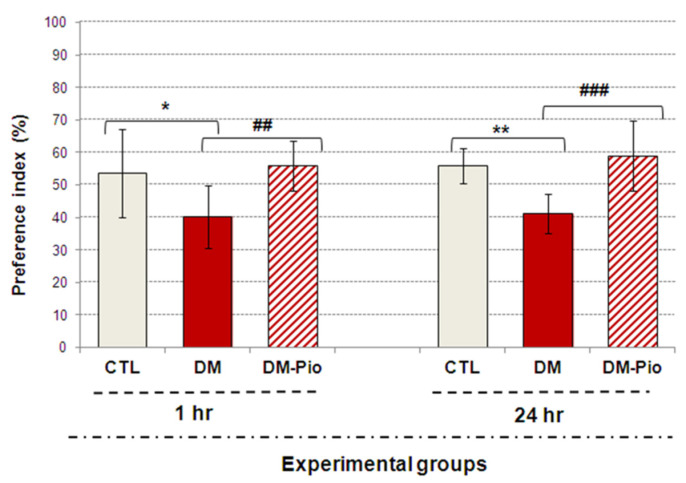
Effect of pioglitazone on impaired memory of mice with neuroinflammation related with diabetes. The bar height represents the mean ± SD for individual experimental groups: CTL: non-diabetic mice, DM: diabetic mice, DM-Pio: pioglitazone-treated mice [30 mg/kg, *po*]. One-way ANOVA, followed by Tukey’s post hoc test was used. * *p* < 0.05; ** *p* < 0.01 in comparison with control group. ## *p* < 0.01; ### *p* < 0.001 in comparison with diabetes group.

As can be seen in Figure 3, in the testing phase after both a 1-h and 24-h break from the end of the training phase, pioglitazone-treated mice showed a remarkable increase in interest in exploring the novel object in comparison with the known one (Figure 3, ## *p* < 0.01, F_(3.32)_ = 7.268; ### *p* < 0.001, F_(3.35)_ = 15.433, respectively). The value of the preference index (%) in treated mice group was significantly above 55%, which indicates that they were able to distinguish between these objects, as opposed to untreated mice. For comparison, the preference index (%PI) in diabetic mice was significantly below 50% (Figure 3, * *p* < 0.05, F_(3.32)_ = 7.268; ** *p* < 0.01, F_(3.35)_ = 15.433, respectively) suggesting that this group of animals had recognition memory deficits. Furthermore, it is worth mentioning that the performed statistical analysis did not indicate the preference of the object in the training stage in none of the experimental groups and mice spent a similar amount of time testing objects (results not shown).

### 2.2. Effect of Pioglitazone on the Level of Peripheral and Central Pro-Inflammatory Cytokines in Diabetes Mice

**Figure 4:**
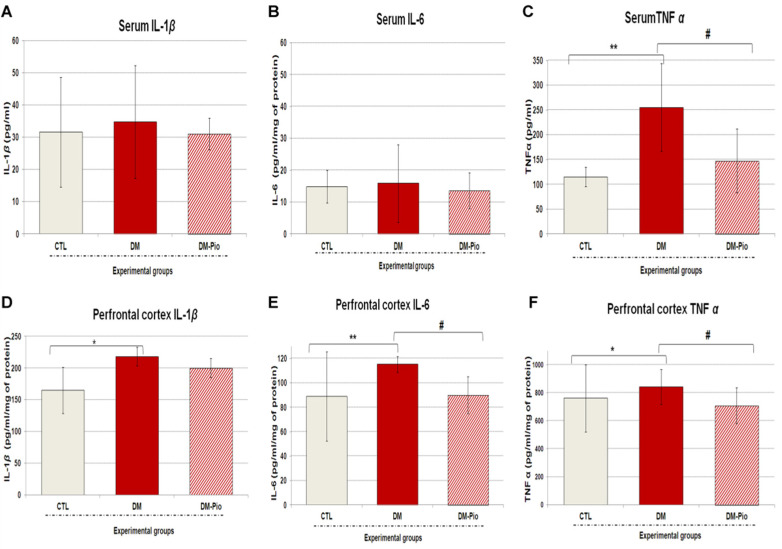
Influence of pioglitazone on inflammatory mediators in the blood serum or prefrontal cortex of diabetic mice. IL-1*β* (**A**), IL-6 (**B**), and TNF*α* (**C**) were measured by ELISA kits in the serum of blood; IL-1*β* (**D**), IL-6 (**E**), and TNF*α* (**F**) were measured by ELISA kits in the prefrontal cortex. All data were analyzed by one-way ANOVA, then Tukey’s *post-hoc* test for comparing differences between non-diabetic mice group (CTL), diabetic group (DM), and pioglitazone-treated group (DM-Pio) [30 mg/kg, *po*]. All data have been presented as mean ± SD. * *p* < 0.05; ** *p* < 0.01 in comparison with control group. # *p* < 0.05 in comparison with diabetes group.

As shown in Figure 4A–C, pioglitazone therapy did not influence the level of peripheral inflammatory mediators, except for the TNF*α* level. As shown in the Figure 4C, the 2-weeks treatment with pioglitazone (30 mg/kg *po*) significantly decreased the level of tumor necrosis factor α in the blood serum of treated mice compared to the diabetic animals (Figure 4C, # *p* < 0.05, F_(3.49)_ = 9.500). In turn, a one-way ANOVA with a Tukey’s post hoc test, as seen in the Figure 4D–F, revealed that the fourteen-day therapy with pioglitazone significantly reduced interleukin 6 and tumor necrosis factor *α* levels in the prefrontal cortex of mice compared to the untreated animals group (by 20 and 16%, respectively; Figure 4E,F, # *p* < 0.05, F_(3.88)_ = 1.86; # *p* < 0.05, F_(3.88)_ = 0.7801). Whereas pioglitazone therapy had no significant effect on the elevated brain level of interleukin 1β in diabetic mice (Figure 4D; *p* > 0.05).

### 2.3. Changes in Tnfrsf1a and Cav1 Gene Expression in the Hippocampus and Prefrontal Cortex of Diabetes Mice in Response to Treatment with Pio

**Figure 5:**
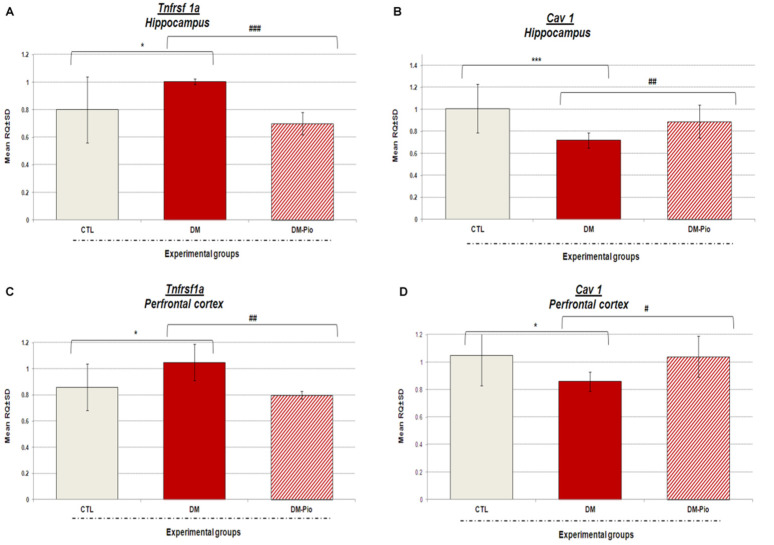
Expression of mRNA levels of *Tnfrsf1a* and *Cav1* in the hippocampus and prefrontal cortex of diabetic mice after 2 weeks treatment with pioglitazone. The bar height represents the mean ± SD for *Tnfrsf1a* and *Cav1* gene expression in individual experimental groups: CTL: non-diabetic mice, DM: diabetic mice, DM-Pio: pioglitazone-treated mice [30 mg/kg, *po*] in the hippocampus (**A**,**C**) or prefrontal cortex (**B**,**D**) of mice. One-way ANOVA, followed by Tukey’s post hoc test was used. * *p* < 0.05; *** *p* < 0.001 in comparison with control group. # *p* < 0.05; ## *p* < 0.01; ### *p* < 0.001 in comparison with diabetes group.

As shown in Figure 5A–D in response to 14 days pioglitazone treatment (30 mg/kg, *po*) there were noted significant downregulations of *Tnfrsf1a* expression and upregulations of *Cav1* expression in the studied brain regions of diabetic mice. *Tnfrsf1a* gene expression was lowered more in the hippocampus than in the prefrontal cortex of treated mice. In the hippocampus, *Tnfrsf1a* expression level was lower by 30% in relation to the mRNA level of this gene in untreated mice (Figure 5A, ### *p* < 0.001, F_(3.31)_ = 10.868). Whereas, in the prefrontal cortex, the down-regulation of *Tnfrsf1a* by 23%, compared to the expression of this gene in diabetic animals (Figure 5C, ## *p* < 0.01, F_(3.32)_ = 8.749), was observed. In turn, the level of *Cav1* expression in the hippocampus and prefrontal cortex in the group of animals treated with pioglitazone was significantly higher than in the group of untreated animals, by 24 and 18%, respectively (Figure 5B–D, ## *p* < 0.01, F_(3.32)_ = 16.775; # *p* < 0.05, F_(3.31)_ = 4.395, respectively). In addition, higher increases in the expression of this gene were observed in the hippocampus than in the prefrontal cortex of mice after treatment with pioglitazone (Figure 5B–D).

### 2.4. Effect of Pioglitazone on Level of Glucose during the Oral Glucose Tolerance Test (OGTT) in Diabetes Mice

**Figure 6:**
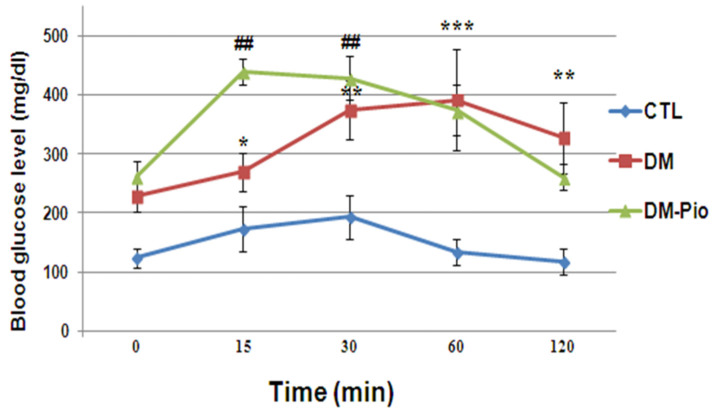
Changes in blood glucose level in oral glucose tolerance test (OGTT) performed in diabetes mice after 2-week treatment with pioglitazone. The blood glucose levels (mg/dl) were measured at different time points using hand-held glucometer. Fasting blood glucose concentration was used as a reference for OGTT. Data were expressed as mean ± SD. Blue line (CTL): non-diabetic mice; red line (DM): diabetic mice; green line (DM-Pio): pioglitazone-treated mice [30 mg/kg, *po*]. One-way ANOVA, followed by Tukey’s post hoc test was used. * *p* < 0.05; ** ## *p* < 0.01; *** *p* < 0.001 compared with baseline level of glucose (before the glucose load).

As shown in Figure 6, diabetes mice showed the abnormal glucose levels before the test. These levels in the diabetes mice were significantly increased at 15, 30, and 60 min after oral glucose administration compared with the baseline level of glucose (2h before the glucose load) (Figure 6, * *p* < 0.05, F_(2.75)_ = 7.738; ** *p* < 0.01, F_(2.75)_ = 7.738; *** *p* < 0.001, F_(2.75)_ = 7.738). Additionally, the results of OGTT showed an extreme glucose intolerance in the above-mentioned group. The basal level of glucose was not attained even after 2 h after the glucose load (Figure 6, ** *p* < 0.01, F_(2.75)_ =7.738). Whereas, 14-day treatment with pioglitazone revealed improvements in glucose tolerance in diabetes mice, as measured by OGTT. As shown in Figure 6, the drug resulted in the return of glycaemia to the baseline values in 2 h after the glucose load.

### 2.5. Statistical Analysis

Statistical analysis was performed using one-way analysis of variance (ANOVA). When significant statistical differences were obtained between the experimental groups, the Tukey’s post hoc test was used. For statistical analysis we used GraphPad Prism software, version 8.0.1 (GraphPad Prism, San Diego, CA, USA). In all the calculations, the significance level was assumed at *p* <  0.05. Results are expressed as the mean  ±  SD.

## 3. Discussion

Our studies have confirmed the presence of neuroinflammation in diabetic mice. Higher levels of neuroinflammatory and molecular indicators in the brains of sick mice were positively correlated with the degree of impairment of their cognitive functions, such as memory and learning. Research has shown that the increased level of pro-inflammatory cytokines disturbs the neurotrophic factor signaling pathway that in turn leads to decreased neurotrophic support and neurogenesis [59,60,61,62]. Ida et al. [63] demonstrated that pro-inflammatory cytokines can lead to the impairment of neurogenesis also through N-methyl-d-aspartate receptor activation. Studies by Gavillet et al. [64] and Thornton et al. [65] showed that activated astrocytes and microglia, apart from the cytokines and chemokines, can also release reactive oxygen and nitrogen species that cause the oxidative damage of neurons. In recent years, many researchers have turned their attention to the role of circulating cytokines in the development of CNS disorders. Studies have shown that chronic inflammation and the associated elevated levels of circulating cytokines often correlate with cognitive decline in patients [66,67]. The pro-inflammatory cytokines released in these disorders may activate the nuclear factor kappa B signaling pathway (NF-kB). NF-kB, a primary transcription factor, promotes the inflammatory response and passes peripheral inflammatory signals to the central nervous system, leading to chronic and progressive neuronal loss [68,69,70]. Our research revealed that diabetes as a metabolic disease not only upregulated the expression of a broad profile of pro-inflammatory cytokines in the prefrontal cortex of mice, but it can also induce systemic inflammation accompanied by a significant increase in serum TNF*α* levels. It is worth mentioning that, both TNF*α* of central and peripheral origins can affect cognitive functions [71]. TNF*α* from the periphery can penetrate the intact blood–brain barrier (BBB) through a transcytosis process mediated by TNF-I as well as TNF-II receptors [72,73]. Besides, TNF*α* can stimulate the expression of IL-1 and IL-6 [74]. Our studies showed that mice with high levels of TNF*α* exhibited impaired short- and long-term memory in the NOR test. They showed no preference for novelty, so they were unable to distinguish between familiar and new objects. It appears that, particularly lesions of the perirhinal cortex, led to the NOR test performance deficit [75,76,77]. We also observed that inflammation had a negative effect on exploratory activity of mice in the open field test (OFT) and the hole-board test (HBT). Furthermore, Roberts et al. [78] showed that mice with an overexpression of IL-6 in the brain are characterized by reduced exploratory activity in a light–dark transfer test and depressive-like behavior in a forced swim test. In turn, Eyre et al. [79] showed that elevated TNF*α* levels cause a reduction in hippocampal volume through the neurodegenerative TNFRSF1A pathway, which can lead to the development of depressive-like behavior. Moreover, a one-way analysis of variance with Tukey’s post hoc test showed that diabetes suppresses the expression of the *Cav1* gene in the hippocampus and the prefrontal cortex of mice, which correlates with the results of behavioral research described earlier [59] and those presented in this paper. Moreover, our results are compatible with the observations of other researchers [80]. Niesman et al. [81] and Head et al. [82] showed that CAV1-null mice have heightened brain lesion volumes, neuroinflammation, and accelerated neurodegeneration. In addition, in the caveolin-1 knockout mice, the neurological abnormalities, as seen in Alzheimer’s disease, were noted [83,84].

Chronic hyperglycemia or poor glycemic control may result in the decreased transport of glucose from the blood to the brain, thereby disrupting its function [32,33]. Patients with diabetes and those with increased non-diabetic fasting glucose levels are more likely to develop cognitive dysfunction than patients with normal glucose metabolism [27,33,34]. Our study confirmed the beneficial effect of pioglitazone on glucose metabolism. We proved that 14-day treatment led to improvements in glucose tolerance in diabetic mice, as measured by OGTT.

Due to the fact that inflammation is a significant factor in the pathogenesis of diabetes as well as the neurological disorders related to this disease, the therapy targeting neuroinflammatory signaling may provide a promising strategy for intervening in the development of CNS disorders.

Some of the most important properties of pioglitazone (apart from the antihyperglycemic effect) are the anti-inflammatory and anti-oxidative effects [46,47,48,85,86,87]. Many recent studies suggest that pioglitazone may have neuroprotective properties [43,46,47,49], which could potentially increase its role not only in diabetes treatment but also in CNS complications. The available studies showed the evidence that PPAR agonists exhibit anti-inflammatory and antioxidant effects in several models of CNS disorders, such as ischemic stroke, Alzheimer’s, and Parkinson’s diseases [43,88]. They also proved that PPAR*γ* agonists may have beneficial effects on the CNS via regulation of cytokine production and/or by reducing oxidative stress [37,38]. Research demonstrated that the PPAR*γ* activation regulates the expression of proinflammatory genes that translates into the reduced secretion of IL-1*β* and IL-6 by monocytes [89,90]. In turn, Baghcheghi et al. [91] showed the protective effects of the PPAR*γ* agonists, rosiglitazone and pioglitazone, in the cerebellar oxidative damage in hypothyroid rats. A study by Masciopinto et al. [92] demonstrated improved cognitive function in a female population of PS1-KI mutant mice with cognitive deficits after a 9-month treatment with pioglitazone. Furthermore, the aforementioned therapy enhanced short-term memory performance in male wildtype (WT) mice with the decreased activity of complex I in hippocampal and cortical mitochondria. Most of the researchers reported that the protective effect of pioglitazone in the brain is dose-dependent, as well as dependent on the length of time that the drug is taken by the patient. However, since only ∼18% of orally administered pioglitazone crosses the intact blood–brain barrier in mammals [93], it seems likely that drug dosage, rather than treatment duration, is critical to observe drug effects in the brain [94]. This is confirmed by the results of Seok et al. [95]. They proved that lower doses of pioglitazone are more effective than the higher ones used for the same duration. SAMP8 mice (representing the behavioral and pathological features of late-onset and age-related sporadic AD) administered with pioglitazone at 2 mg/kg/day for 7 weeks, showed fewer Aβ deposits and reduced A*β*1-40 levels, increased LRP1 expression, and significant improvements in cognitive function. In turn, Grommes et al. [96] studied the transport of pioglitazone across the BBB and they proved that this drug administered orally can cross the barrier. Authors verified the ability of the drug to cross the BBB by measuring its concentration in the brain parenchyma.

Our research showed that pioglitazone therapy had little effect on the levels of peripheral inflammatory mediators in diabetic mice. There was only a significant reduction in the level of tumor necrosis factor α observed in the blood serum of treated diabetic mice compared to untreated ones. However, we noted the significant downregulation of the *Tnfrsf1a* gene expression in both the hippocampus and the prefrontal cortex, as well as the reduction in the level of proinflammatory proteins such as TNF*α* and IL-6 in the prefrontal cortex of diabetic mice. It is also worth noting that the *Tnfrsf1a* gene expression was lowered more in the hippocampus than in the prefrontal cortex of treated mice. This must be highlighted because crucial cognitive abilities such as spatial memory are linked just with the hippocampus.

An extensive literature review implicates that synaptic plasticity in the acquisition, consolidation, and long-term storage of different types of memory may depend on the levels of cytokines and gene expressions such as caveolin-1 (*Cav1*) [46,97]. The in vitro studies by Wang et al. [98] showed that the overexpression of *Cav1* in murine macrophages dramatically inhibited TNF*α* and IL-6 production but increased the secretion of the anti-inflammatory interleukin 10. We demonstrated that a central blockade of pro-inflammatory cytokine synthesis as well as *Tnfrsf1a* gene expression, but an upregulation of *Cav1* mRNA level caused by pioglitazone, led to significant improvements in cognitive functions. Diabetes mice that received pioglitazone for 14 days showed an amelioration in short- and long-term memory outcomes measured by the NOR test. Moreover, in the open field test and the hole-board test, we noticed that there was an improvement of impaired exploratory activity in treated mice. The available research shows that significant changes in mRNA levels of TNFRSF1A in the brain may be associated with the modulation of the pro-inflammatory NF-κB signaling pathway [98]. Various studies indicated that compounds targeting the above-mentioned pathway as ligand/receptor, adapter protein, or transcription factors may have anti-inflammatory, neuroprotective, or analgesic effects [99,100,101,102,103,104]. A study by Deng et al. [105] showed that pioglitazone may effectively reduce the neuroinflammation by the PPAR*γ*/NF-κB/IL-6 signaling pathway. El-Sahar et al. [43] confirmed the antioxidant and anti-inflammatory action of pioglitazone (10 mg/kg, *po*) administered daily for 2 weeks in diabetic rats with ischemia/reperfusion (I/R) injury. This drug, by suppressing the nuclear factor kappa (NFκB), led to decreased levels of pro-inflammatory cytokines, such as tumor necrosis factor α and interleukin-6, and to increased levels of anti-inflammatory cytokines such as interleukin-10. Additionally, the drug reduced oxidative stress by influencing the level of reduced glutathione, nitric oxide, and lipid peroxidation. Furthermore, a study by Yamagishi et al. [106] confirmed the anti-inflammatory effect of pioglitazone in experimental diabetic neuropathy in rats. Treatment with pioglitazone for 12 weeks (10 mg/kg/day orally) reduced the disruption of the PKC pathway and the pro-inflammatory process. It is believed that pioglitazone could decrease protein oxidation and lipid peroxidation in the brain, while it also increases antioxidant capabilities of the brain. These findings give important evidence to prove that the production of pro-inflammatory proteins and gene expression can be effectively controlled by pioglitazone. It is particularly important, due to the fact that the neuroinflammation seems to underlie the development of various disorders of the central nervous system and diseases, such as Alzheimer’s disease and Parkinson’s disease. Furthermore, the pharmacological control of glucose metabolism may have a significant impact on limiting the progression of cognitive disorders in the group of patients with impaired glucose tolerance and/or diabetes.

## 4. Materials and Methods

### 4.1. Animals and Treatments

Adult, CD-1 male mice (output weight of 20–24 g) were obtained from a licensed breeder (Experimental Medicine Centre (EMC), Medical University of Lublin, Lublin, Poland, (077—EMC number in Lublin in the Breeders’ Register kept by the Minister of Science and Higher Education, (Lublin, Poland)). Diabetes type 2 was induced as previously described [59]. In short, mice (7 to 8 weeks old) were randomly divided into groups. Diabetes was induced in mice by *ad libitum* administration of 20% aqueous fructose solution for 4 weeks and after that, for the next 5 days (1× daily), the animals were intraperitoneally injected with freshly prepared solution of STZ (40 mg/kg of body weight, *ip*) in 0.01 M cold citrate buffer pH 4,5. Control group mice (CTL) were administered with citrate buffer only. The total number of animals was estimated in accordance with the requirements of statistical analyses, the Three Rs (3Rs) and the ARRIVE guidelines (Animal Research: Reporting of In Vivo Experiments).

In the next stage of the experiment, one of the groups of animals with confirmed T2M was received one dose of antidiabetic drug-pioglitazone (30 mg/kg in an amount depending on the individual’s body weight: 0.05 mL/10g body weight) daily, for 14 days *per os* (gastric tube). At the same time, mice from the remaining two groups (CTL-control; DM-diabetes) received physiologic saline (the same route of administration). Each group consisted of eight male mice. At the end of the experiment, mice were killed by decapitation and blood for clot and brains were collected, as previously described [59].

All procedures were approved by the Local Ethics Committee on Animal Experimentation in Lublin (No. 43/2018) and were performed in accordance with binding European standards related to the experimental studies on animal models (Act from 15 January 2015 on the Protection of Animals Used for Scientific or Educational Purposes; Directive 2010/63/eu of the European Parliament and of the council of 22 September 2010 on the protection of animals used for scientific purposes). All mice were housed in a room with automatically controlled temperature (22 ± 2 °C), relative humidity (45–65%), and light-dark (12/12 h) cycles.

Twenty-four hours after administration of the last dose of pioglitazone, the mice were subjected to behavioral tests evaluating their exploratory behaviors, and memory in the following order: the open-field test (OFT), the hole-board test (HBT), and novelty object recognition test (NOR test). The same tests were carried out on animals in the remaining experimental groups.

### 4.2. Behavioral Tests

#### 4.2.1. Open Field Test (OFT)

The open field test (OFT) was used to assess the exploratory behaviors activity of mice. The test was carried out to the procedure described by Hall [107] in the box constructed of natural wood with the floor dimensions of 40 × 40 cm and a wall height of 35 cm. Mice were individually placed at the center of the arena and allowed to explore the arena, freely and uninterrupted, for 5 min. The assessment of exploratory activity was based on measuring the time the animals moved (s) in the arena. As well as recording the time spent by the mice in the central part of the arena and in the periphery of the arena.

#### 4.2.2. The Hole-Board Test (HBT)

The hole-board test (HBT) was used to assess the exploratory activity of mice. The device was made up of a black plastic plate with a size of 35 × 35 cm^2^, and 5 cm thick, with evenly distributed 16 circular holes, each measuring 28 mm in diameter. The apparatus was elevated from the floor to a height of 25 cm. Each mouse was individually placed at the center of the plate and allowed to explore the arena freely. During 5 min of observation, the apparatus automatically counted the number of head dips of mice into the holes. The measure of exploratory activity was the total number of head dips of mice into the holes in a given experimental group. The test was according to the method described by Boissier and Simon [108,109]. The cage and blocks were thoroughly cleaned between each use with 70% ethanol.

#### 4.2.3. The Novel Object Recognition Test (NOR)

The novel object recognition (NOR) test is used to evaluate cognition, particularly memory. This test is based on the natural tendency of animals to spend more time exploring a novel object than a familiar one. The test was carried out in three stages (habituation, training, and testing) according to the previously described procedure [59]. The test objects were wooden blocks in the shape of an oval, rectangular, or triangular pyramid (4 cm × 4 cm × 6 cm). During 10 min habituation session, the animals were placed in the arena and they were allowed to freely explore an empty box. After twenty-four hours, the mice were placed again in the arena with two identical oval white blocks—training stage. During 5 min of observation, the number of interactions of the animal with each of the objects was recorded. Following a delay (after 1 h as well as 24 h), an analogous testing session was performed, but one of the blocks was replaced by a new, as yet unknown object. During 5 min of observation, the number of exploring behaviors of each object by a mouse was recorded. NOR test performance was quantified using the Preference Index (%PI). %PI is calculated as TN/(TN + TF) × 100%, where TN corresponds to the exploration time of the novel object (Nov) whereas TF corresponds to exploration time of the familiar (Fam) object. Preference index at the level of 50% reflects equal exploration of novel and familiar objects. During the training phase, the time spent exploring the two objects by animals in all groups was similar (*p* > 0.05, results not shown).

### 4.3. Collection of Biological Materials for Research

After performing the behavioral tests, the animals were sacrificed by decapitation, and the biological material was collected. Blood was taken into tubes that did not contain an anticoagulant in order to obtain serum. The brains were carefully removed from the skull and then, immediately, the prefrontal cortex and hippocampus were separated. Serum and brain tissues were directly frozen in −80 °C.

#### 4.3.1. Measurement of Cytokines in the Serum and Brain Supernatants

The collected blood was allowed to clot and then it was centrifuged at 1000× *g* for 10 min. Then, serum samples were collected into clean tubes and stored at −20 °C until used in ELISA assays. The isolated brain prefrontal cortexes were homogenized and lysed using a lysis buffer (Cloud-Clone Corp., Houston, TX, USA), following the manufacturer’s directions. Lysates were centrifuged at 10,000 rpm and 4 °C for 5 min and then they were stored at −80 °C until used. Protein content was determined by Bradford method [110]. The levels of IL-1*β*, IL-6, and TNF*α*, were quantified in a single serum or brain tissue sample using an appropriate for mice ELISA Kit (Cloud-Clone Corp., Houston, TX, USA), as previously described [59]. We performed the determinations in accordance with the manufacturer’s instruction. The optical density of individual wells was measured with a spectrophotometric microplate reader (BioTek, Elx808, Warsaw, Poland) at a wavelength of 450 nm. The concentration of cytokine in the samples was determined by comparing the optical density of the samples with the standard curve. Cytokine concentrations in the prefrontal cortex are expressed in picograms per ml/mg of protein, and picograms per ml of serum.

#### 4.3.2. The Quantitative Real-Time PCR Analysis (qRT-PCR)

##### RNA Isolation from Hippocampus and Prefrontal Cortex of Mice

Total RNA was isolated from the hippocampus and prefrontal cortex of mice by the method of Chomczyński and Sacchi [111] using the TRIzol reagent (Invitrogen, Carlsbad, CA, USA) according to the manufacturer’s instructions. RNA concentration and purity was measured spectrophotometrically with a MaestroNano NanoDrop spectrophotometer (Maestrogen, Hsinchu, Taiwan). For further analyses, high purity RNA was used (A260/280 ratio between 1.8 and 2.0).

##### CDNA Synthesis

CDNA synthesis was performed using a cDNA reverse transcription kit (Applied Biosystems, Foster City, California, USA) according to the manufacturer’s instructions. The reaction was performed under the following conditions: 25 °C for 10 min, 37 °C for 120 min, and then 85 °C for 5 min to complete the process. The obtained cDNA was stored at −20 °C.

##### Real-Time PCR

In the experiment, the relative expression of the *Tnfrsf1a* and *Cav1* genes was measured, using *Hprt* and *Tbp* as endogenous controls (Table 1). The reaction was performed in triplicate using the 7500 Fast Real-Time PCR System (Applied Biosystems, Foster City, CA, USA) and Fast Probe qPCR Master Mix (2x) plus ROX solution (EURx, Gdańsk, Poland). The reaction mixture contained 10 µL of Fast Probe qPCR Master Mix (2x), 9 µL of RNase-free water, ROX solution (50 nM), and 0.5 µM of the gene-specific TaqMan probe (Applied Biosystems, Foster City, CA, USA). Data quality screening was performed based on amplification, Tm and Ct values to remove any outliers before calculating ΔΔCt and determining the fold change in mRNA levels. The data is presented as an RQ value (RQ = 2 − ΔΔCt).

The primer sequences, gene symbols, assay IDs, gene names, and amplicon lengths (bp) for the genes analyzed are presented in Table 1.

#### 4.3.3. Oral Glucose Tolerance Test (OGTT)

The oral glucose tolerance test was performed in mice that were fasted for 6 h before a single oral dose of glucose solution (2 g/kg body weight) was administered using gavage. Immediately prior to the administration of the glucose solution, animal fasting blood glucose was determined in the blood taken from the tail vein by portable glucometer ACCU-CHEK (Roche, Mannheim, Germany). Next, the blood samples were collected at 15, 30, 60, and 120 min after the glucose load to measure the glucose levels.

## 5. Conclusions and Prospects for the Future

Neuroinflammation is one of the potential mechanisms mediating the onset of a broad range of CNS disorders. This study demonstrates that pioglitazone effectively reduces the neuroinflammation in diabetic mice by reducing the levels of pro-inflammatory cytokines in the brain and modifying the genes expression. Modulating inflammation by pioglitazone was related with the improvement of impaired exploratory activity and the restoration of memory in treated mice. These results suggest that the aforementioned drug may be a promising therapeutic approach for the treatment of neuroinflammation. Our study has also offered some hints on the potential mechanisms of neuroprotective action of pioglitazone. Because we could not measure markers related to oxidative stress or the NF-κB pathway, we cannot exclude the possibility of modulation of those processes by pioglitazone. Future investigation will be necessary to better understand the molecular and cellular mechanisms underlying the therapeutic action of pioglitazone in the neuroinflammation-associated disease pathogenesis.

## Figures and Tables

**Table 1:** The table shows the data on used primers: Gene symbols, assay IDs, gene names, GenBank reference sequence accession numbers, and amplicon lengths (bp).

Gene Symbol	Assay ID	Gene Name	RefSeq	Amplicon Length (bp)
***Tnfrsf1a***	AB ID: Mm00441883_g1	Tumor necrosis factor receptor superfamily, member 1a	NM_011609.4	82
***Cav1***	AB ID: Mm00483057_m1	Caveolin 1	NM_001243064.1 NM_007616.4	67
***Hprt***	AB ID: Mm00446968_m1	Hypoxanthine guanine phosphoribosyl transferase	NM_013556.2	65
***Tbp***	AB ID: Mm00446974_m1	TATA box binding protein	NM_013684.3	105

AB ID, Applied Biosystems TaqMan Gene Expression Assay ID.

## Data Availability

Not applicable.
